# Systematic analysis of brain and skull ischemic injury expression profiles reveals associations of the tumor immune microenvironment and cell death with ischemic stroke

**DOI:** 10.3389/fimmu.2022.1082546

**Published:** 2022-12-20

**Authors:** Chao Zhang, Lisi Wang, Yunmiao Guo, Wei Feng

**Affiliations:** ^1^Zhanjiang Institute of Clinical Medicine, Central People’s Hospital of Zhanjiang, Zhanjiang, Guangdong, China; ^2^Zhanjiang Central Hospital, Guangdong Medical University, Zhanjiang, Guangdong, China

**Keywords:** ischemic stroke, cell death, tumor, immune microenvironment, skull, brain

## Abstract

**Background:**

Previous studies have shown that stroke is a potential first sign of neoplasia, but the relationship between stroke and cancer remains unclear. As a complex brain disease, ischemic stroke involves cell death and immunity. Thus, it is necessary to investigate the association of the tumor immune microenvironment and cell death with ischemic stroke.

**Methods:**

We established a photothrombosis-induced ischemic injury model in mouse brain and skull. Subsequently, we sequenced the whole transcriptome of the injured mouse brain and skull and analyzed the expression profiles. To investigate the association of stroke with cell death and cancer, we systematically performed gene set enrichment analysis in pan-cell death (i.e., apoptosis, cuproptosis, ferroptosis, necroptosis, and pyroptosis) and the cancer hallmark pathways. The time-dependent immune cell abundance variations after ischemic injury were estimated. Furthermore, pan-cancer genomic and prognostic analyses of the ischemic injury-related gene sets were also performed.

**Results:**

In this study, we found that there exist temporal and spatial differences in the gene expression patterns of both the brain and skull with ischemic injury. The skull ischemic injury-induced changes in the brain transcriptome were particularly great, but could recover in a short period, while the skull transcriptome variation resulting from brain ischemic injury was long-lasting. In addition, the expression of the genes related to ischemic injury was also associated with pan-cell death and the cancer hallmark pathways. The changes in the abundance of immune cells indicate that brain ischemic injury may disrupt the immune microenvironment for a longer time, while the skull can balance the stability of the immune microenvironment better. Moreover, the brain ischemic injury-related gene sets were highly correlated with a variety of tumors, particularly glioblastoma multiforme (GBM), kidney renal clear cell carcinoma (KIRC), brain lower grade glioma (LGG), and uveal melanoma (UVM), which carry a greater mortality risk after stroke.

**Conclusion:**

This systematic analysis not only helps in the understanding of the changes in the gene expression profiles of both the brain and skull with ischemic injury but also reveals the association of the tumor immune microenvironment and cell death with ischemic stroke.

## 1 Introduction

Cancer and stroke are among the leading causes of death worldwide (1–3) that share various epidemiological risk factors and bring a huge public health burden. Stroke, a heterogeneous pathological process, will lead to acute neurological damage. A previous study illustrated that cancer is one of the potential risk factors associated with stroke (4). Cerebrovascular disease is the second most common neurological manifestation after metastasis in cancer patients (5). In addition, a previous study reported that about 15% of patients with cancer had a stroke, half of whom were asymptomatic (6). A recent study has also shown that the risk of stroke among patients with cancer was twice that of the general population and increases with longer follow-up (7). In addition, patients diagnosed with a brain tumor face a high risk of stroke throughout life (7). This means that patients with cancer have a high risk of stroke (8–12). Stroke may be a potential first sign of neoplasia. However, the relationship between cancer and stroke is still unclear.

Recent studies have challenged our cognition of the skull. Findings indicate that the skull bone marrow plays an important role in immunosurveillance of the central nervous system (CNS) (13, 14). Some studies have reported a direct local vascular connection between the brain and the skull bone marrow through the meninges (14–16). Additionally, a significant increase in the number of cranial myeloid cells that migrate to the meninges and injury area after stroke onset has been observed (7). This means that the skull is an important component of brain immunity that plays an essential role in the process of cerebral ischemic injury and repair. However, the changes that occur in the skull after brain ischemic injury and the effects of skull damage on the brain remain to be elucidated. Some early studies discovered *via* transcriptome analysis that many differential gene enrichments after ischemic stroke are associated with cancer (17). As is known, severe hypoxia occurs in the area of brain lesions after ischemic stroke. Hypoxia is a widespread trait in 90% of solid tumors, which has a profound effect on cell proliferation, metabolism, migration, and angiogenesis during cell development and disease (18–20). Therefore, it is necessary to investigate the associations between ischemic injury to the brain/skull and the tumor.

In addition, cerebral ischemia could result in secondary brain injury and neuronal death, producing inflammatory mediators and leading to immune responses in brain tissues (21). After ischemic stroke, the damaged cells could cause systemic immune response by releasing the specific signals that activate immunodepression (22). These studies all suggest that immune response and inflammation are important factors associated with the pathogenesis of stroke and its outcomes (23, 24). In recent years, a growing number of studies have shown that ischemic stroke is a complex brain disease regulated by multiple cell death pathways, including apoptosis, necroptosis, and ferroptosis (25, 26). However, the relevant mechanisms still need to be explored further. Until just recently, cuproptosis has been newly characterized as a form of cell death (27); whether it is associated with ischemic stroke has not been reported yet.

In this work, a photothrombosis-induced ischemic injury model in the brain and skull of mice was established. The gene expression profiles of the brain and skull after ischemic injury were analyzed and compared with the whole transcriptome. Enrichments of the relevant differential genes in the cancer and cell death pathways were also explored. Moreover, changes in the immune microenvironment after ischemic injury were further analyzed. Finally, pan-cancer genomic and prognostic analyses of the ischemic injury-related gene sets were performed.

## 2 Materials and methods

### 2.1 Animal preparation and ethics

All experimental procedures were performed according to the animal experiment guidelines of the Experimental Animal Management Ordinance of Guangdong Province, China, and the guidelines from the Guangdong Medical University, which have been approved by the Institutional Animal Ethics Committee of Central People’s Hospital of Zhanjiang. Wild-type male BALB/c mice (*n* = 33, 8 weeks old) were used for ischemic injury modeling. The mice were raised under specified pathogen-free (SPF) conditions, with a 12/12-h light/dark cycle.

### 2.2 Photothrombosis ischemic injury model

A mouse focal photothrombosis (PT) ischemic injury model was constructed after establishing the optical clearing skull window. About 100 μl of Rose Bengal (RB) solution (4 mg/ml; Sigma-Aldrich, St. Louis, MO, USA) was injected *via* the tail vein right before laser irradiation, and occlusion was induced with 532 nm laser (1 mm diameter, 2 mW; Laserwave, Beijing, China) irradiation for 210 s. When the light spot was controlled to 1 mm in diameter, the light source was about 2 cm away from the head of the mouse.

### 2.3 Assessment of brain injury with rhodamine extravasation

Mice in the control, PT-2 (2 days after skull PT), and PT-6 (6 days after skull PT) groups were injected with 0.05 ml rhodamine (0.5 mg/ml; Sigma-Aldrich, St. Louis, MO, USA) through the tail vein. About 30 min after circulation, the mice were decapitated and their brain rapidly taken out and fixed in 4% paraformaldehyde (PFA) for 24 h. Subsequently, the brains were cut into 50 μm slices and stained with DAPI. Images of the intact brain slices were captured using a fluorescence microscope (Ni-E Microscope; Nikon, Tokyo, Japan) at ×20 objective (NA = 0.75). ImageJ software was employed to analyze the images.

### 2.4 Transcriptome profiling

Mouse brain and skull tissues with the ischemic and peri-ischemic regions were harvested for RNA sequencing (RNA-seq) at representative time points. Each group of skull or brain tissue samples was kept in nearly the same volume.

### 2.5 RNA-seq library preparation

The total RNA of the samples was extracted using TRIzol reagent (Thermo Fisher Scientific Inc., Waltham, MA, USA) and further treated with DNase to remove contamination of the genomic DNA. The messenger RNA (mRNA) was isolated using the NEBNext Poly(A) mRNA Magnetic Isolation Module (New England Biolabs, Ipswich, MA, USA), which was then used for the preparation of the RNA-seq library with the NEBNext Ultra II mRNA Library Prep Kit for Illumina (New England Biolabs, Ipswich, MA, USA). These were performed using Illumina sequencing with the paired-end 2 × 150 sequencing mode.

### 2.6 RNA-seq data quality control and preprocessing

Raw RNA-seq data in FASTQ format were processed with fastp v0.23.0 (28), an ultra-fast FASTQ preprocessor with useful quality control and data filtering using default parameters. Afterward, the clean reads were mapped to the *Mus musculus* genome (mm10) with HISAT2 v2.1.0 (29). To integrate our previous RNA-seq data into the current work, we used ComBat_seq from the R package sva (v3.42.0) (30) to remove batch effects. Differential expression analysis of two groups (three biological replicates per condition) was performed using the R package DESeq2 (v1.18.1) (31). Normalized counts were derived using DESeq2 to compare the gene expression across samples (32). Genes with |log2(fold change)| >1 and a false discovery rate (FDR) <0.05 were considered as differentially expressed genes (DEG).

### 2.7 Gene set enrichment and variation analyses for the RNA-seq data

The Gene Ontology (GO) term enrichment and the gene set enrichment analysis (GSEA) (33) between groups were performed with the R package clusterProfiler (v4.0) (34) with default parameters on the MSigDB (35) C5 and hallmark pathways. The gene set variation analysis (GSVA) scores were estimated using the R package GSVA (v1.42.0) (36) for the gene set in each sample. We used ComplexHeatmap v2.10.0 (37) to generate heatmaps.

### 2.8 Estimation of immune cell abundance

The immune cell abundance was estimated based on the expression read count data of each sample with ImmuCellAI-mouse (Immune Cell Abundance Identifier for mouse) (38). Significant differences in the immune cell abundance values between the injured groups and the control groups were analyzed with the two-sided Student’s *t*-test. A *p*-value less than 0.05 was considered to indicate significant difference. We used the Kruskal–Wallis test to determine the statistically significant differences between three groups. For the correlation between gene expression and immune cell abundance, we used Pearson’s correlation and considered |*R*| > 0.9 and *p* < 0.05 to represent a significant correlation.

### 2.9 Statistical analysis

All figures herein without specification were plotted with the R package ggplot2. The *p*-values of the other tests without specification were calculated using a two-sided Student’s *t*-test. *P*-values less than 0.05 were considered statistically significant.

## 3 Results

### 3.1 Transcriptome analysis of brain and skull ischemic injury

**Figure 1:**
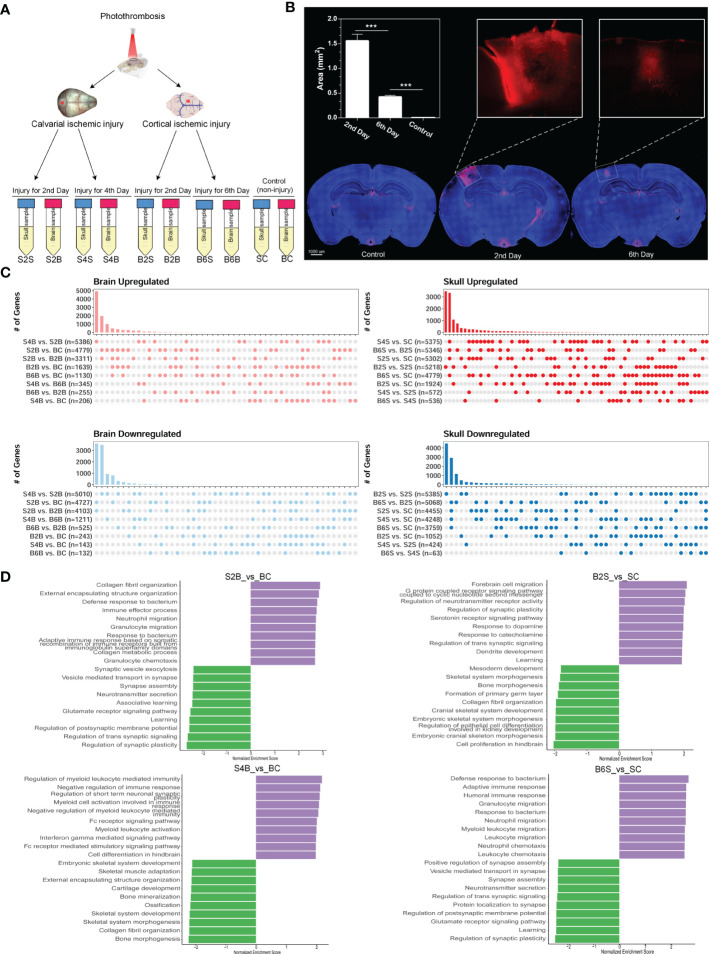
Experimental design and transcriptome analysis of the brain and skull after photothrombosis (PT). **(A)** Experimental design of the brain and skull PT. *BC*, control group for the uninjured brain; *B2B* and *B6B*, brain samples of the second and the sixth day after brain PT; *SC*, control group for the uninjured skull; *S2S* and *S4S*, skull samples of the second and the fourth day after skull PT; *B2S* and *B6S*, skull samples of the second and the sixth day after brain PT; *S2B* and *S4B*, brain samples of the second and the fourth day after skull PT. **(B)** Rhodamine extravasation evaluating the degree of brain injury at different time points after brain PT (*n* = 3, mean ± standard deviation). ***p < 0.001. **(C)** Number of up- and downregulated genes between the brain and the skull groups. **(D)** Gene set enrichment analysis (GSEA) of brain and skull ischemic injury with adjusted *p*-values less than 0.05 (top 10 up- and downregulated terms).

Here, we established the ischemic injury model using PT for the brain and skull and performed whole-transcriptome sequencing, including the injured skull (second and fourth days), the skull with brain injury (second and sixth days), the injured brain (second and sixth days), the brain with skull injury (second and fourth days), and the skull and brain in the control groups (**Figure 1A**). The time points for the sample selection referred to our previous work (39). In order to verify the effectiveness of model establishment and to evaluate brain recovery after ischemic injury, we evaluated the changes in the blood–brain barrier after ischemic injury from the acute phase (second day) to the chronic phase (sixth day) based on the area of dye leakage (**Figure 1B**). The results showed that the disruption of the blood–brain barrier was severe on the second day after PT, but was greatly restored on the sixth day after PT. Moreover, we combined the transcriptome data of the untreated brain and skull as control groups from our previous work (39) (GSE191183). We performed removal of batch effects for the transcriptome dataset before further analysis, as shown in **Supplementary Figure S1**. Subsequently, we determined the DEGs for brain and skull sequencing, as shown in **Supplementary Figures S2A, B** and **Table S1**. We further investigated the changes in the number of DEGs in the brain after skull and brain injury. By comparing with the control group, we found that the number of upregulated and downregulated genes in the brain on the second day after skull PT (S2B vs. BC: 4,779 upregulated and 4,727 downregulated) was more than that on the second day after brain PT (B2B vs. BC: 1,639 upregulated and243 downregulated). On the contrary, the number of up- and downregulated genes in the brain 4 days after skull PT (S4B vs. BC: 206 upregulated and143 downregulated) was less than that at 6 days after brain PT (B6B vs. BC: 1,130 upregulated and 132 downregulated). Interestingly, compared with the brain after skull and brain PT (second day), the number of up- and downregulated genes in the brain after skull PT (fourth day) (S4B vs. S2B: 5,386 upregulated and 5,010 downregulated) was far more than that after brain PT (sixth day) (B6B vs. B2B: 255 upregulated and 525 downregulated) (**Figure 1C**, left panel). However, we observed different phenomena in the skull after skull and brain injury. Compared with the control group, the number of up- and downregulated genes in the skull after brain PT (second day) (B2S vs. SC: 1,924 upregulated and 1,052 downregulated) was less than that after skull PT (second day) (S2S vs. SC: 5,302 upregulated and 4,455 downregulated). But the number of up- and downregulated genes in the skull after brain PT (sixth day) (B6S vs. SC: 4,779 upregulated and 3,759 downregulated) was less than that after skull PT (fourth day) (S4S vs. SC: 5,375 upregulated and 4,248 downregulated). Compared with the skull after brain and skull PT (second day), the number of up- and downregulated genes in the skull after brain PT (sixth day) (B6S vs. B2S: 5,346 upregulated and 5,068 downregulated) was far more than that after skull PT (fourth day) (S4S vs. S2S: 572 regulated and 424 downregulated) (**Figure 3C**, right panel).

Furthermore, GSEA was performed for S2/4B vs. BC and B2/6S vs. SC. For the brain on the second day after skull PT, the upregulated biological processes were mostly associated with the immune effector process and immune cell migration, whereas the processes related to regulation of synapse and neurotransmitter transport were downregulated (S2B vs. BC) (**Figure 1D**, top left panel). For the brain on the fourth day after skull PT, the upregulated biological processes were mostly enriched in the regulation of immune response and myeloid cell activation, while the downregulated biological processes mainly included skeletal system development and ossification (S4B vs. BC) (**Figure 1D**, bottom left panel). For the skull on the second day after brain PT, the processes associated with forebrain cell migration, regulation of synapse, and neurotransmitter activity were upregulated, while the downregulated biological processes were mostly enriched in skeletal system development and bone morphogenesis (B2S vs. SC) (**Figure 1D**, top right panel). For the skull on the sixth day after brain PT, the processes related to immune response and immune cell migration were upregulated, and most of the biological processes on cell communication were downregulated (B6S vs. SC) (**Figure 1D**, bottom right panel). These results indicate the existence of close immune communications between the brain and the skull after ischemic injury; thus, researchers should pay more attention to the immune response of the skull in brain disease studies.

**Figure 2:**
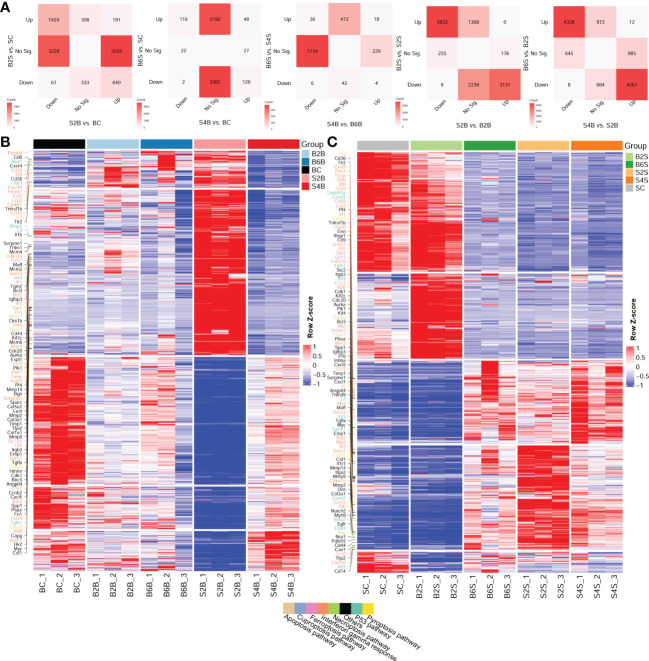
Comparative analysis of the up- and downregulation of the differentially expressed genes (DEGs) between the brain and skull with ischemic injury at different time points. **(A)** Number of intersecting DEGs in the different groups of brain and skull samples (upregulated: FC > 2 and FDR < 0.05; downregulated: FC < 1/2 and FDR < 0.05). *FC*, fold change; *FDR*, false discovery rate*; No Sig.*, not significant. **(B, C)** Heatmap of the brain **(B)** and the skull **(C)** after photothrombosis (PT) with DEGs (*different colored genes* are the representative genes from the cancer hallmark to the cell death pathways). *BC*, control group for the uninjured brain; *B2B* and *B6B*, brain samples of the second and sixth days after brain PT; *SC*, control group for the uninjured skull; *S2S* and *S4S*, skull samples of the second and fourth days after skull PT; *B2S* and *B6S*, skull samples of the second and sixth days after brain PT; *S2B* and *S4B*, brain samples of the second and fourth days after skull PT. (**B**, **C**, show the rows clustered using Pearson’s correlations).

Furthermore, we compared the DEGs between the brain and the skull after ischemic injury, as shown in **Figure 2A**. For the brain on day 2 after skull PT (vs. BC) and the skull on day 2 after brain PT (vs. SC), 3,228 genes were downregulated only in the brain, which were mostly related to the regulation of synapse and neurotransmitter transport (**Supplementary Figure S3** and **Table S2**). A total of 3,558 genes were upregulated only in the brain, and most of these genes were related to extracellular matrix organization and wounding healing. In addition, 308 genes were upregulated only in the skull, with the enriched GO terms including muscle contraction, organ morphogenesis, and cell motility. For the brain on day 4 after skull PT (vs. BC) and the skull on day 6 after brain PT (vs. SC) (**Supplementary Figure S3** and **Table S2**), 3,382 genes were downregulated only in the skull, most of which were associated with the regulation of synapse and neurotransmitter transport. Moreover, 3,192 genes were upregulated only in the skull, which were mainly involved in extracellular matrix organization and cell migration. For the brain on day 4 after skull PT (vs. B6B) and the skull on day 6 after brain PT (vs. S4S) (**Supplementary Figure S3** and **Table S2**), 1,134 genes were downregulated only in the brain, which were mainly involved in extracellular matrix organization and immune response. There were 229 genes upregulated only in the brain, most of them associated with neurotransmitter transport and neural activity. A total of 472 genes were upregulated only in the skull, most of which were related to regulation of synapse and neurotransmitter transport.

We also investigated the expression patterns of the DEGs across the different time points after ischemic injury of the brain and skull, shown in the heatmaps in **Figures 2B, C**. We found that, for the brain, the expression patterns of the DEGs changed dramatically on day 2 after skull PT compared to those of the brain on days 2 and 6 after brain PT, but this difference almost disappeared by day 4 after skull PT.

For the skull, the expression patterns of the DEGs were greatly altered on day 2 after skull PT compared to the control groups, which did not recover until day 4. However, the expression patterns of the DEGs in the skull changed slightly on day 2 after brain PT, with the major changes occurring on day 6. For both brain and skull ischemic injury, we found that a large number of the gene expression changes among these groups were related to cancer hallmark and cell death pathways, including the P53, interferon gamma response, pyroptosis, necroptosis, ferroptosis, cuproptosis, and apoptosis pathways.

### 3.2 Associations between brain/skull ischemic injury and cell death

**Figure 3:**
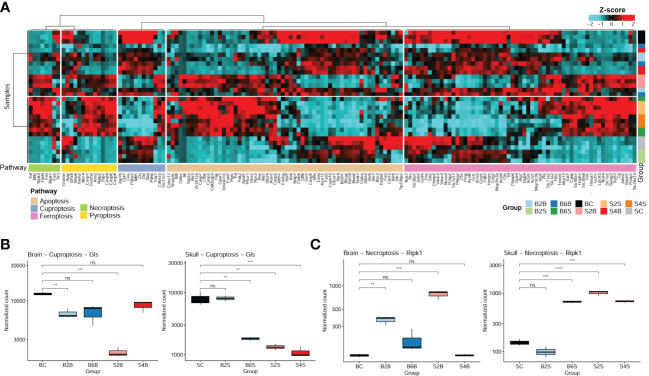
Expression patterns of the cell death pathway genes in brain and skull ischemic injury. **(A)** Heatmap of the genes in the cell death pathways (apoptosis, cuproptosis, ferroptosis, necroptosis, and pyroptosis pathways) for brain and skull ischemic injury. **(B, C)** Analysis of the changes in the representative genes *Ripk1* **(B)** and *Gls* **(C)** associated with necroptosis and cuproptosis in brain and skull ischemic injury (*n* = 3). *ns*, not significant. **p < 0.01, ***p < 0.001, ****p < 0.0001. *BC*, control group for the uninjured brain; *B2B* and *B6B*, brain samples of the second and sixth days after brain photothrombosis (PT); *SC*, control group for the uninjured skull; *S2S* and *S4S*, skull samples of the second and fourth days after skull PT; *B2S* and *B6S*, skull samples of the second and sixth days after brain PT; *S2B* and *S4B*, brain samples of the second and fourth days after skull PT. (**A**, the “cluster_within_group” function was used for the row and column clusters based on Pearson’s correlation).

Cell death is one of the most important mechanisms in the development of ischemic stroke (40). Here, we systematically analyzed the changes in the gene expression profiles related to five cell death pathways for the brain and skull in different groups, including the injured skull (days 2 and 4), the skull with brain injury (days 2 and 4), the injured brain (days 2 and 6), the brain with skull injury (days 2 and 6), and the skull and brain in the control groups. We found that ischemic injury of the brain and skull was associated with the cell death pathways of apoptosis, ferroptosis, cuproptosis, necroptosis, and pyroptosis (**Figure 3A**), and the genes involved in apoptosis and ferroptosis accounted for the majority. In addition, the gene expression profiles related to cell death pathways varied at different injured sites and ischemic injury time points.

We found that the expression of the cuproptosis-related genes (CRGs) (27, 41) (i.e., *Gls*, *Atp7b*, *Cdkn2a*, *Dlat*, *Dld*, *Fdx1*, *Lias*, *Lipt1*, *Mtf1*, *Pdha1*, *Pdhb*, and *Slc31a1*) changed dramatically between brain and skull ischemic injury. Intriguingly, *Gls* is involved in ischemic stroke-related ferroptosis (42) and has also been identified as a typical cuproptosis-associated gene (43). We systematically evaluated the genetic alterations of the *Gls*-related cuproptosis pathway in the different experimental groups, as shown in **Figure 3B**. On the second day after brain PT, *Gls* in the damaged brain tissue was significantly downregulated (*p* < 0.01), which returned to normal on the sixth day. Moreover, *Gls* in the brain after skull PT was also significantly downregulated on the second day (*p* < 0.001), also returning to normal on the fourth day. On the second day after brain PT, *Gls* in the skull hardly changed, but was significantly downregulated on the sixth day (*p* < 0.01). As for the second and fourth days after skull PT, *Gls* in the damaged skull tissue was significantly downregulated (*p* < 0.01).

It has been shown that early ischemic brain injury activates *Ripk1* in endothelial cells to drive the necroptosis process in order to induce cerebral hemorrhagic phenomena and pro-inflammatory responses. Thus, we also analyzed changes of the necroptosis-related typical gene (*Ripk1*) in brain and skull ischemic injury (**Figure 3C**). On the second day after brain PT, *Ripk1* in the damaged brain tissue was significantly upregulated (*p* < 0.01), which returned to normal on the sixth day. The *Ripk1* gene in the brain after skull PT was also significantly upregulated on the second day (*p* < 0.001) and returned to normal on the fourth day. On the second day after brain PT, the *Ripk1* gene in the skull was hardly changed, but was significantly upregulated on the sixth day. On the second and fourth days after skull PT, *Ripk1* in the damaged skull tissue was significantly upregulated (*p* < 0.001). It has been widely reported that stroke leads to changes in the *Gls* and *Ripk1* genes (42, 44, 45), which is consistent with the changes in the expression levels of *Gls* and *Ripk1* in the brain on the second day after brain injury (B2B vs. BC). These results indicate a close association between cell death and brain/skull ischemic injury, which might play an important role in ischemic stroke.

### 3.3 Association of ischemic injury with cancer hallmark pathways

**Figure 4:**
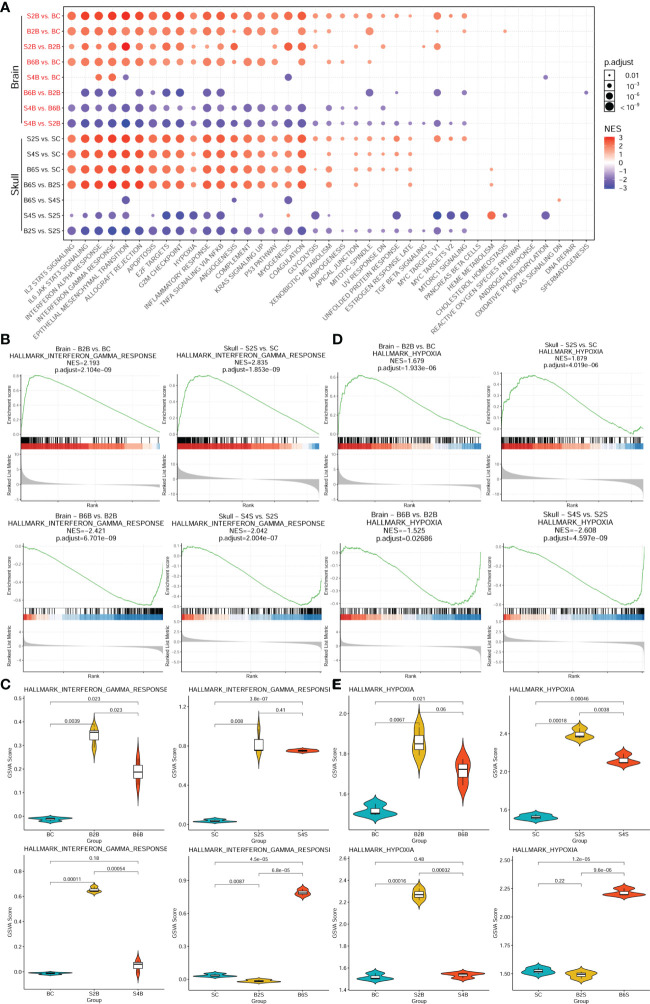
Enrichment analysis of the genes associated with the cancer hallmark pathways in brain and skull ischemic injury. **(A)** Enrichment patterns of the brain and skull ischemic injury-related genes in the cancer hallmark pathways. **(B, D)** Gene set enrichment analysis (GSEA) between the brain and skull ischemic injury groups for the representative interferon gamma response **(B)** and hypoxia **(D)** pathways. **(C, E)** Gene set variation analysis (GSVA) scores of the single samples for the representative interferon gamma response **(C)** and hypoxia **(E)** pathways (*n* = 3). *BC*, control group for the uninjured brain; *B2B* and *B6B*, brain samples of the second and sixth days after brain photothrombosis (PT); *SC*, control group for the uninjured skull; *S2S* and *S4S*, skull samples of the second and fourth days after skull PT; *B2S* and *B6S*, skull samples of the second and sixth days after brain PT; *S2B* and *S4B*, brain samples of the second and fourth days after skull PT.

We further performed enrichment analysis of the cancer hallmark gene sets of the different groups after brain and skull ischemic injury, as shown in **Figure 4** and **Supplementary Table S3**. There were 39 cancer hallmark pathways associated with brain and skull ischemic injury. **Figure 4A** shows the consistent pattern of variations in the up- and downregulation of the gene expression in these cancer hallmark pathways (e.g., IL2/STAT5 signaling, IL6/JAK/STAT3 signaling, interferon alpha response, interferon gamma response, epithelial–mesenchymal transition, allograft rejection, apoptosis, E2F targets, G2M checkpoint, hypoxia, inflammatory response, TNF-α signaling, NF-κB, angiogenesis, complement, KRAS signaling, P53 pathway, and myogenesis). The pattern of alterations in the brain after brain PT was found to be almost identical to that in the skull after skull PT (B2B vs. BC, B6B vs. BC, S2S vs. SC, and S4S vs. SC), and a higher enrichment score of the skull groups was also found. On the second day after skull PT, the brain showed evident changes (S2B vs. BC), which almost returned to the normal pattern by the day 4 (S4B vs. BC). However, on the second day after brain PT, the brain showed almost no changes (B2S vs. SC), but significant changes appeared on the sixth day (B6S vs. SC). Furthermore, two representative cancer hallmark pathways (i.e., interferon gamma response and hypoxia) were selected to explore the changes in brain and skull ischemic injury based on the GSEA plots (**Figures 4B, D**). The results showed a consistent pattern of changes between brain and skull ischemic injury, but manifested completely opposite patterns of change at different time points after ischemic injury.

Moreover, comparisons of the GSVA scores representing the extent of interferon gamma response and hypoxia between the brain and skull ischemic injury groups showed that the genes associated with both pathways were significantly upregulated on day 2 after ischemic injury (*p* < 0.01) (**Figures 4C, E**) and were downregulated in the injured sites on days 4 and 6. However, after skull PT, the genes related to these two pathways in the brain were significantly upregulated on the second day (*p* < 0.001), but returned to normal on the fourth day. On the other hand, the genes related to these two pathways did not change significantly in the skull tissues on the second day after brain PT (*p* > 0.05), but significant changes occurred on the sixth day. These results suggest that the effect of skull ischemic injury on the brain manifests rapidly, while there is a relative delay in the effect of brain ischemic injury on the skull.

### 3.4 Alterations in the immune cell abundance during brain and skull ischemic injury

**Figure 5:**
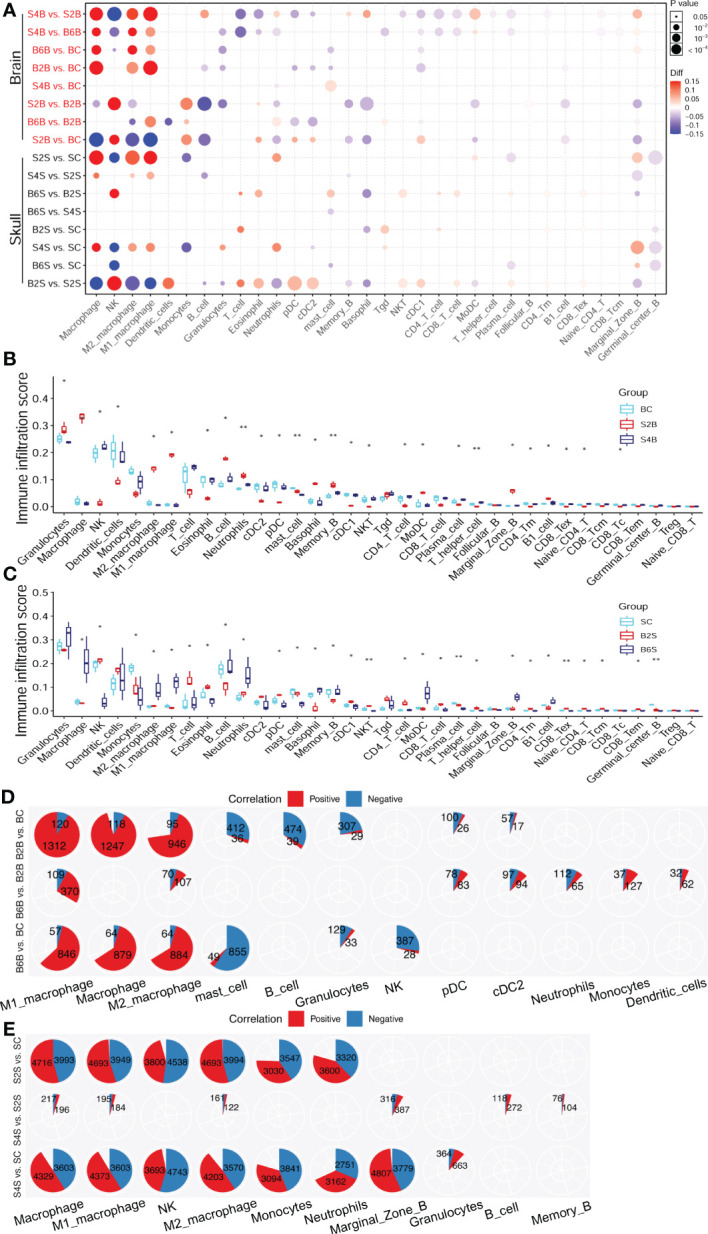
Changes in the immune cell abundance after ischemic injury. **(A)** Immune abundance changes in the brain and skull groups. **(B, C)** Boxplot of the immune cell abundance differences in the brain **(B)** and skull **(C)** groups (*n* = 3; Kruskal–Wallis test: *p < 0.05, **p < 0.01). **(D, E)** Positive (*red*) and negative (*blue*) correlation patterns between the ischemic injury-related genes and the different immune cell types across the brain **(D)** and skull **(E)** ischemic injury groups (Pearson’s correlation: *r* > 0.9, *p* < 0.05). *BC*, control group for the uninjured brain; *B2B* and *B6B*, brain samples of the second and sixth days after brain photothrombosis (PT); *SC*, control group for the uninjured skull; *S2S* and *S4S*, skull samples of the second and fourth days after skull PT; *B2S* and *B6S*, skull samples of the second and sixth days after brain PT; *S2B* and *S4B*, brain samples of the second and fourth days after skull PT.

We further analyzed the association of immune cell abundance with brain and skull ischemic injury. We determined the immune cells from bulk RNA-seq data using the immune cell abundance estimation tool ImmuCellAI-mouse and found 32 classes of immune cells altered during brain and skull ischemic injury, as shown in **Supplementary Table S4**. **Figure 5A** shows that, on the second day after skull PT, the immune cell abundance in the brain was altered, but recovered by the fourth day. In contrast, on the second and sixth days after brain PT, the immune cell abundance in the skull had little alteration. This indicates that the skull possesses a relatively stable immune microenvironment and plays a very important role in brain immunity. Additionally, we predicted the abundance of immune cells, which revealed dynamic changes in immune cell infiltration during brain and skull ischemic injury (**Figures 5B, C**). We found that the immune microenvironment of the brain was indeed affected by skull injury on the second day, whereas the brain almost returned to normal on the fourth day. However, the immune cell abundance in the skull was not changed immediately when ischemic injury occurred in the brain, but was activated on the sixth day. In order to understand the molecular mechanisms of the alterations in the immune cell abundance after brain and skull ischemic injury, the correlation between the brain and skull ischemic injury-related genes and immune cells needs to be unveiled.

The number of immune cell infiltration-related genes after brain ischemic injury was less than that after skull ischemic injury (**Figures 5D, E**). The abundance of macrophages increased significantly on the second day after brain ischemic injury (*p* < 0.05). A total of 1,312 genes were positive for M1 macrophage, while only 120 genes were negative. Similarly, 946 genes were positive for M2 macrophage, while only 95 genes were negative. For day 6 of brain ischemic injury, 846 genes were positive for M1 macrophage abundance, but only 57 genes were negative. Similarly, 884 genes were positive for M2 macrophage abundance, but only 64 genes were negative (**Figure 5D**). The abundance of macrophages was significantly increased on the second day after skull ischemic injury (*p* < 0.05), with 4,693 genes positive and 3,949 genes negative for M1 macrophage abundance. Similarly, 4,693 genes were positive and 3,994 genes were negative for the abundance of M2 macrophage. For day 4 of skull ischemic injury, 4,373 genes were positive and only 3,603 genes were negative for M1 macrophage abundance. Similarly, 4,203 genes were positive and only 3,570 genes were negative for M2 macrophage abundance (**Figure 5E**). Furthermore, the number of genes with positive and negative correlations were roughly similar in each immune cell for skull ischemic injury, whereas for there was a huge gap between the number of positive and negative correlations for brain ischemic injury. These results suggest that ischemic injury-related genes are associated with alterations in the abundance of immune cells. We can reasonably speculate that brain ischemic injury may disrupt its immune microenvironment for a longer time, while the skull can balance the stability of the immune microenvironment better.

### 3.5 Pan-cancer genomic and prognosis analyses of ischemic injury-related genes

**Figure 6:**
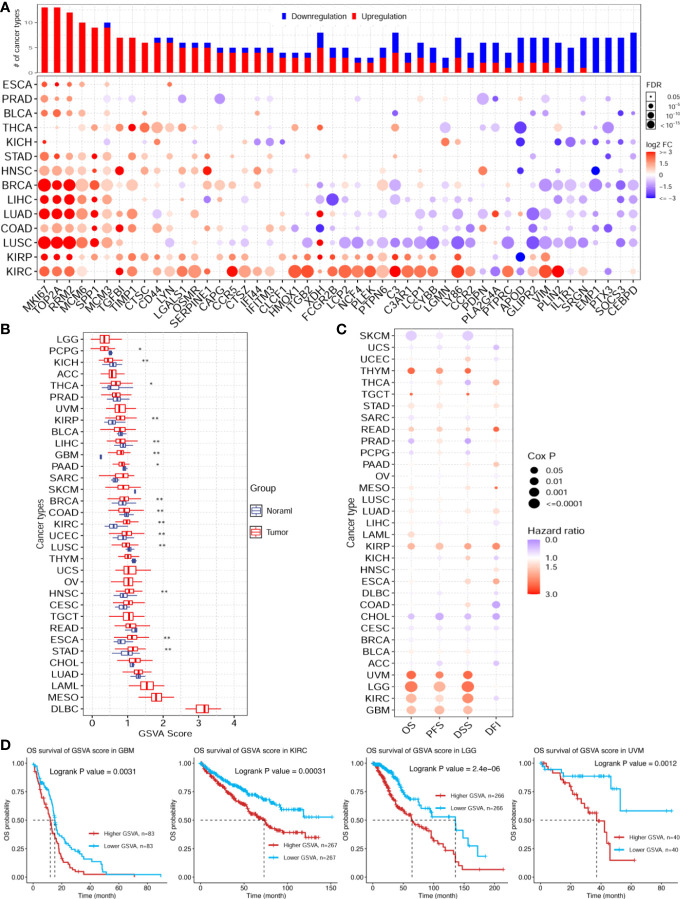
Pan-cancer genomic and prognostic analyses of the ischemic injury-associated genes. **(A)** Upregulation (*red*) and downregulation (*blue*) patterns of the ischemic injury-associated gene sets across different cancer types (*y*-axis) compared to paired normal samples. The *x*-axis represents the 50 core brain ischemic injury-associated genes. The *color intensity* indicates the fold change, while the *point size* indicates the significance of the *p*-value. *Upper bars* show the frequency of cancer types, with upregulation (*red*) and downregulation (*blue*) for each brain ischemic injury-related gene. **(B)** Gene set variation analysis (GSVA) scores of the ischemic injury-associated gene set in each cancer type (*n* = 3). *p < 0.05, **p < 0.01. **(C)** Expression of the ischemic injury-associated genes correlated with the overall survival (OS), progression-free survival (PFS), disease-specific survival (DSS), and disease-free survival **(DFI)** in various cancers from The Cancer Genome Atlas (TCGA). **(D)** Kaplan–Meier curves showing the OS of the ischemic injury-related gene sets analyzed in glioblastoma multiforme (GBM), kidney renal clear cell carcinoma (KIRC), brain lower grade glioma (LGG), and uveal melanoma (UVM). *BC*, control group for the uninjured brain; *B2B* and *B6B*, brain samples of the second and sixth days after brain photothrombosis (PT); *SC*, control group for the uninjured skull; *S2S* and *S4S*, skull samples of the second and fourth days after skull PT; *B2S* and *B6S*, skull samples of the second and sixth days after brain PT; *S2B* and *S4B*, brain samples of the second and fourth days after skull PT.

The enrichment analysis showed that the ischemic injury DEGs were highly enriched in the cancer hallmark pathways, hinting at the connection between stroke and cancer. To investigate this potential association, we used the top 50 highly expressed genes in the injured brain tissue as the brain ischemic injury-associated genes (BIIGs). Subsequently, we used a gene set cancer analysis (GSCA) web server (46) to explore the genomic and prognostic associations of the BIIGs with The Cancer Genome Atlas (TCGA) pan-cancers (47). We utilized 14 cancer types from TCGA with paired tumor-normal RNA-seq samples to identify the DEGs for BIIGs. Overall, the BIIGs were significantly differentially expressed in 14 cancer types (FDR < 0.05). We interpreted an increased expression as upregulation and a decreased expression as downregulation compared with the control groups (**Figure 6A**). Some BIIGs were upregulated in multiple cancer types: *Top2a*, *Mki67*, *Rrm2*, *Mcm6*, and *Spp1* were upregulated in more than eight cancer types, while *Emp1*, *Ptx3*, and *Socs3* were upregulated in seven cancer types. Some BIIGs were downregulated in various cancers, especially *Il1r1*, *Srgn*, *Emp1*, *Ptx3*, *Socs3*, and *Cebpd*, which were downregulated in at least seven cancer types. A recent study has shown that *Spp1*, *Top2a*, and *Mki67* played a vital role in the inflammatory response during brain ischemic injury (48, 49) and that *Ptx3* was an important target in brain ischemic injury and possibly other brain inflammatory disorders (50). Some other findings indicated that the downregulation of *Socs3* in the microglia/macrophages could lead to a marked bias toward the M2 phenotype and ameliorate the inflammation, which could promote neuroprotective effects after stroke (51). Therefore, this suggests that ischemic injury-associated genes play different roles in different tumor environments.

Furthermore, we evaluated the GSVA scores of the 50 BIIGs in TCGA cancer types, as indicated in **Figure 6B**. We found that the GSVA scores of the BIIGs were low in brain-related tumors [e.g., brain lower grade glioma (LGG) and glioblastoma multiforme (GBM)] and higher in diffuse large B-cell lymphoma (DLBC). Subsequently, we analyzed the association of the BIIGs with prognosis, including overall survival (OS), progression-free survival (PFS), disease-specific survival (DSS), and disease-free survival (DFI), in TCGA cancer types (**Figure 6C**). We classified the patients into a high-BIIG and a low-BIIG group based on the median GSVA scores of the BIIGs. The hazard ratio greater than 1 of the higher GSVA score group compared to the lower GSVA score group indicated a higher risk. We found that tumor patients with GBM, kidney renal clear cell carcinoma (KIRC), LGG, and uveal melanoma (UVM) had a greater mortality risk with higher BIIG GSVA scores. As shown in **Figure 6D**, patients with high expression levels of the ischemic injury-associated genes showed significantly worse survival in GBM (log-rank test, *p* = 0.0031), KIRC (log-rank test, *p* = 0.0031), LGG (log-rank test, *p* = 2 × 10^−6^), and UVM (log-rank test, *p* = 0.0012).

## 4 Discussion

In this study, we evaluated the high GSVA scores of the BIIGs in DLBC, mesothelioma (MESO), and acute myeloid leukemia (LAML) (**Figure 6B**). These highly correlated tumors were not directly related to the brain, which may be due to the limited number of ischemic injury-associated genes we selected for analysis (top 50 highly expressed genes). However, there have also been some case reports showing the strong association between DLBC (52) and MESO (53) and stroke. Additionally, a recent study has demonstrated that RNA signatures in the blood can be used to identify cancer-associated acute ischemic stroke and to monitor patients with cryptogenic acute ischemic stroke for occult cancer (54). This may explain our finding of a higher association between LAML and stroke.

Among the top genes we selected that were consistently overexpressed after brain ischemic injury, *Top2a* has been reported to accelerate tumor development and progression in many cancers, whose upregulation correlated with tumor metastasis and shorter survival in patients (55). Moreover, *Mki67* plays an important role in tumor microenvironment and congenital immunity (56). A lot of studies have reported that *Mki67*, *Rrm2* (57), and *Mcm6* (58) could act as prognostic biomarkers in several cancer types. In addition, *Ptx3* is also an inflammatory molecule related to cancer proliferation, invasion, and metastasis (59). These results fully indicate a certain relationship between the tumor microenvironment and ischemic stroke.

The process of ischemic stroke is inevitably accompanied by cell death due to the cutoff of the nutrient supply to the injured site. Previous stroke-related studies have focused on apoptosis (40). In this work, we found that ischemic injury may involve multiple pathways of cell death, including the apoptosis, cuproptosis, ferroptosis, necroptosis, and pyroptosis pathways. An understanding of the mechanisms of the different cell death pathways is crucial to helping with modern treatments of protecting apoptotic neurons after ischemic stroke.

In this work, the ischemic injury modeling was consistent with our recent study (39), and the time points we selected for brain and skull sampling were based on the vascular regeneration patterns reported. Since we needed to compare and analyze the changes in the brain and skull after ischemic injury, we controlled the degree of ischemic injury; thus, the PT-induced ischemic injury model was quite suitable.

In summary, transcriptome analysis was performed to analyze and compare the gene expression profiles between the brain and the skull after ischemic injury. There exist temporal and spatial differences in the gene expression patterns for both the brain and the skull with ischemic injury, with the skull playing an important role in cerebral ischemic injury. Moreover, the brain ischemic injury-related gene sets were highly correlated with a variety of tumors, particularly GBM, KIRC, LGG, and UVM, which carry a greater mortality risk after stroke.

## Data availability statement

The datasets presented in this study can be found in online repositories. The names of the repository/repositories and accession number(s) can be found below: https://www.ncbi.nlm.nih.gov/geo/, GSE213429; https://figshare.com/, 10.6084/m9.figshare.21203201; https://www.jianguoyun.com/p/DTSdzIgQkf-KCxjy_eEEIAA, Jianguoyun/Nutstore. The RNA-seq data are deposited in the Gene Expression Omnibus database with accession number GSE213429. The code of processing these data and statistical analysis can be accessed on GitHub https://github.com/FengWeiLab/Stroke_cancer_immune.

## Ethics statement

The animal study was reviewed and approved by the Institutional Animal Ethics Committee of Central People’s Hospital of Zhanjiang.

## Author contributions

WF and YG contributed to the research design and performance, data acquisition, data analysis, and manuscript writing. LW and CZ prepared the samples. CZ and YG contributed to the research design, manuscript writing, and sample and data analysis. All authors contributed to the article and approved the submitted version.

## Supplementary material

Supplementary Figure 1 The batch effect removal of RNA-seq data. (BC: control group for uninjured brain; B2B and B6B: brain samples group for the 2^nd^ or 6^th^ day after brain PT; SC: control group for uninjured skull; S2S and S4S: skull samples group for the 2^nd^ or 4^th^ day after skull PT; B2S and B6S: skull samples group for the 2^nd^ or 6^th^ day after brain PT; S2B and S4B: brain samples group for the 2^nd^ or 4^th^ day after skull PT)

Supplementary Figure 2 Volcano plot of DEGs for the brain and skull ischemic injury (up-regulated in purple, down-regulated in green). (BC: control group for uninjured brain; B2B and B6B: brain samples group for the 2^nd^ or 6^th^ day after brain PT; SC: control group for uninjured skull; S2S and S4S: skull samples group for the 2^nd^ or 4^th^ day after skull PT; B2S and B6S: skull samples group for the 2^nd^ or 6^th^ day after brain PT; S2B and S4B: brain samples group for the 2^nd^ or 4^th^ day after skull PT)

Supplementary Figure 3 GO term of biological process enrichment with intersected genes in each group (Top 20 of terms). (BC: control group for uninjured brain; B2B and B6B: brain samples group for the 2^nd^ or 6^th^ day after brain PT; SC: control group for uninjured skull; S2S and S4S: skull samples group for the 2^nd^ or 4^th^ day after skull PT; B2S and B6S: skull samples group for the 2^nd^ or 6^th^ day after brain PT; S2B and S4B: brain samples group for the 2^nd^ or 4^th^ day after skull PT)

Supplementary Table 1 Differential expression genes for each group.

Supplementary Table 2 GO biological process enrichment for up- and down-regulated genes.

Supplementary Table 3 GSVA score of cancer hallmarks for each sample.

Supplementary Table 4 Estimated immune cell abundances for each sample.
