# Supplementary figures and images for: Systematic analysis of brain and skull ischemic injury expression profiles reveals associations of the tumor immune microenvironment and cell death with ischemic stroke
Source: Front Immunol. 2022 Dec 20;13:1082546. doi: 10.3389/fimmu.2022.1082546 (PMC9809284; doi:10.3389/fimmu.2022.1082546)

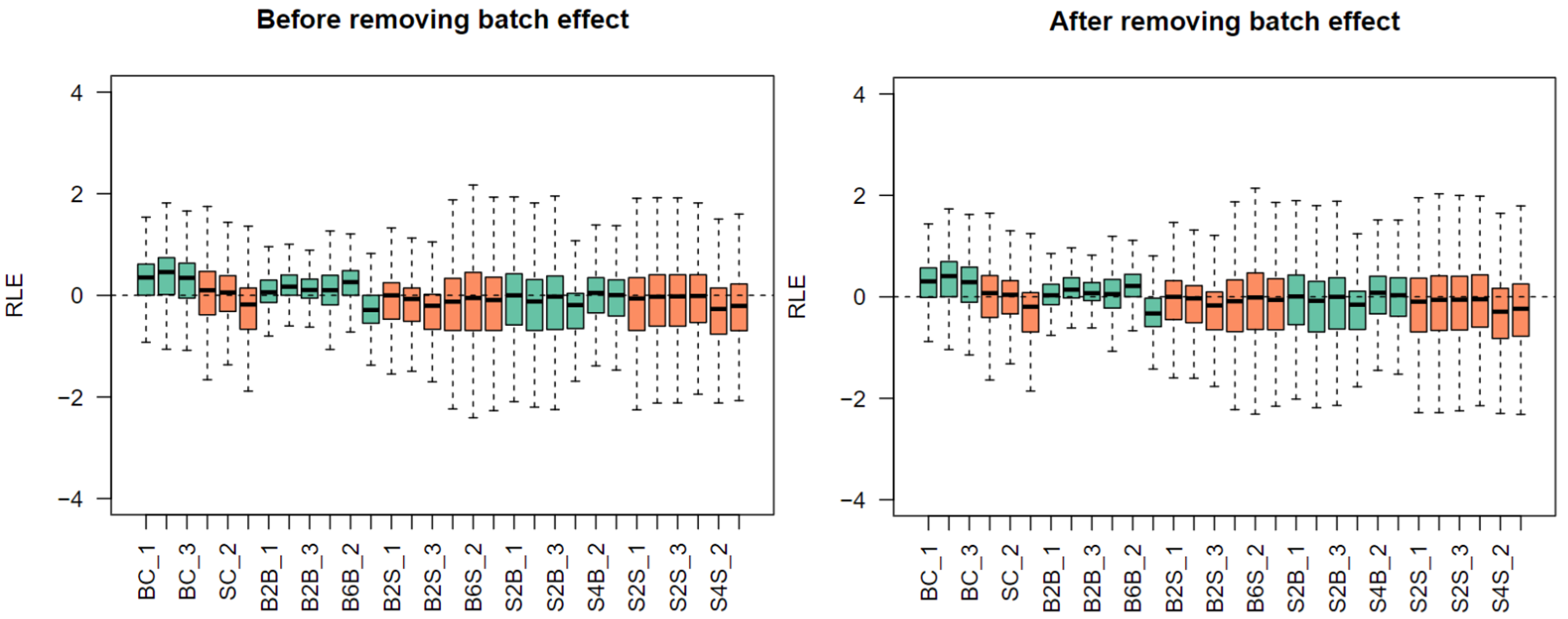

Supplement: Supplementary Figure 1 — The batch effect removal of RNA-seq data. (BC: control group for uninjured brain; B2B and B6B: brain samples group for the 2nd or 6th day after brain PT; SC: control group for uninjured skull; S2S and S4S: skull samples group for the 2nd or 4th day after skull PT; B2S and B6S: skull samples group for the 2nd or 6th day after brain PT; S2B and S4B: brain samples group for the 2nd or 4th day after skull PT) [file Image_1.tif]

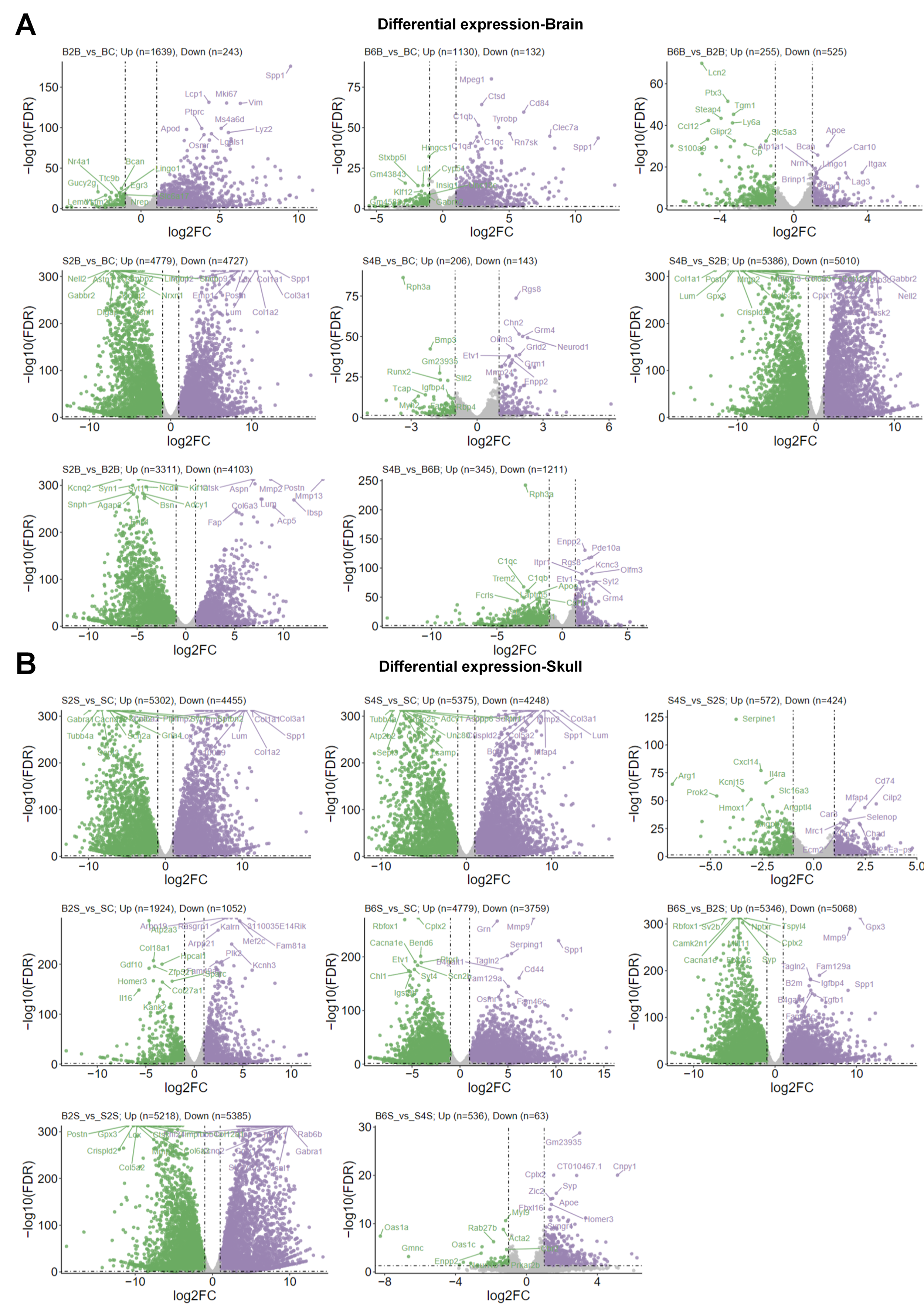

Supplement: Supplementary Figure 2 — Volcano plot of DEGs for the brain and skull ischemic injury (up-regulated in purple, down-regulated in green). (BC: control group for uninjured brain; B2B and B6B: brain samples group for the 2nd or 6th day after brain PT; SC: control group for uninjured skull; S2S and S4S: skull samples group for the 2nd or 4th day after skull PT; B2S and B6S: skull samples group for the 2nd or 6th day after brain PT; S2B and S4B: brain samples group for the 2nd or 4th day after skull PT) [file Image_2.tif]

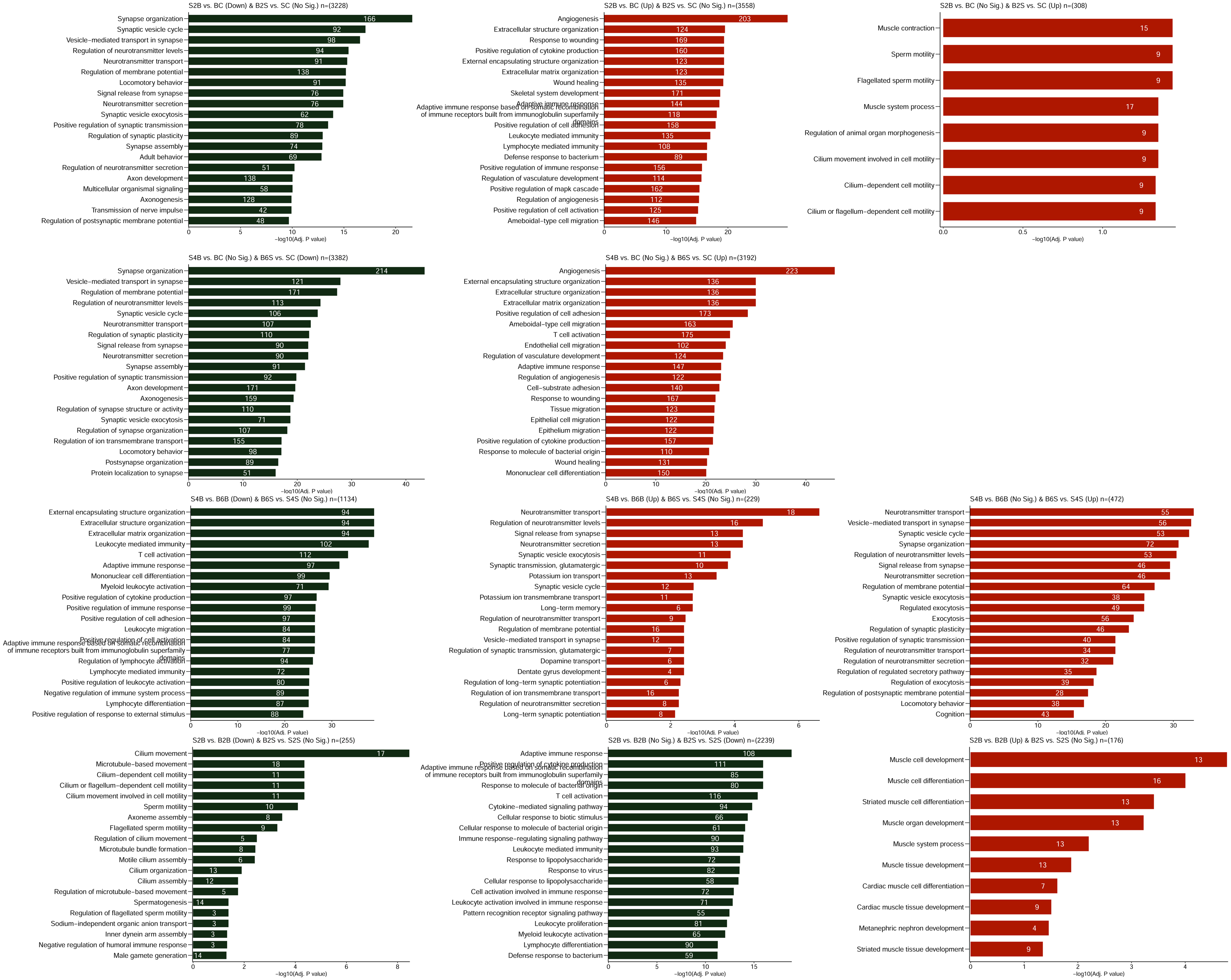

Supplement: Supplementary Figure 3 — GO term of biological process enrichment with intersected genes in each group (Top 20 of terms). (BC: control group for uninjured brain; B2B and B6B: brain samples group for the 2nd or 6th day after brain PT; SC: control group for uninjured skull; S2S and S4S: skull samples group for the 2nd or 4th day after skull PT; B2S and B6S: skull samples group for the 2nd or 6th day after brain PT; S2B and S4B: brain samples group for the 2nd or 4th day after skull PT) [file Image_3.tif]
